# Minimizing Tissue Injury and Incisions in Multilevel Biportal Endoscopic Spine Surgery: Technical Note and Preliminary Results

**DOI:** 10.3390/medicina60030514

**Published:** 2024-03-21

**Authors:** Seung-Kook Kim

**Affiliations:** Department of Spine Center, SNU Seoul Hospital, Gonghangdae-ro 237, Gangseo-gu, Seoul 08703, Republic of Korea; deux8888@naver.com; Tel.: +82-2-333-5151

**Keywords:** biportal endoscopic spinal surgery, endoscopic spine surgery, unilateral biportal endoscopy, degenerative spinal stenosis

## Abstract

*Background and Objectives*: Biportal endoscopic spine surgery (BESS) is a promising technique that can be applied for the treatment of various spinal diseases. However, traditional BESS procedures require multiple, separate incisions. We present, herein, various techniques to reduce the number of incisions in multi-level surgery and their clinical outcomes. *Materials and Methods*: Three different techniques were used to reduce the number of incisions for the preservation of normal tissue associated with BESS: the step-ladder technique, employing a common portal for the scope and instruments; the portal change technique employing a two-level procedure with two incisions; and the tilting technique, employing more than three levels. Pain (Visual Analog Scale), disability (Oswestry Disability Index), and patient satisfaction were evaluated before and 12 months after the procedure. *Results*: Among the 122 cases of multilevel spine surgery, 1.43 incisions per level were employed for multilevel BESS. Pain and disability showed significant improvement. Patient satisfaction showed favorable results. *Conclusions*: Combining multiple techniques during biportal surgery could decrease the number of incisions needed and preserve musculature with favorable clinical outcomes.

## 1. Introduction

Percutaneous biportal endoscopic spine surgery (BESS) is a variant of minimally invasive surgery using separate channels, including both endoscopic and instrumental channels. BESS preserves the musculature and is minimally invasive in nature, which contributes to a short hospital stay and results in operative pain [1,2]. Compared to the uniportal system, separate channels have many advantages, such as stable vision and the free modulation of instruments, facilitating the widening of the spinal canal and easy fusion procedure [3,4]. This technique demonstrated comparable radiological outcomes to those of microscopic and uniportal endoscopic surgery [5]. However, the need for an additional incision is considered disadvantageous in terms of cosmetic outcomes and muscle injury when compared with the uniportal technique. In principle, single-level laminectomy in uniportal endoscopic spine surgery requires a single incision, and BESS requires two incisions. This means that two-level laminectomy requires four incisions, causing twice as much tissue injury than uniportal systems, which represents an avoidable disadvantage of this system. To overcome this, biportal surgeons have developed techniques for preserving muscle and lowering the number of incisions; however, technical illustrations and results have not been reported on this. Thus, we described, herein, various techniques to reduce the number of channels in multi-level surgery and their clinical outcomes.

## 2. Materials and Methods

### 2.1. Indications

This study involved a retrospective analysis of multilevel procedures with a demonstration of the processes using BESS. Written informed consent was obtained from all the patients and the Institutional Review Board approved our study (Approval number: 216390-01-202004-02). The inclusion criteria were as follows: (1) radiologically confirmed spinal canal pathology, (2) unremitting leg radiating pain or neurogenic intermittent claudication (>30 min) receiving conservative treatment for at least 6 weeks, and (3) at least a two-level BESS technique conducted. Exclusion criteria were as follows: (1) extra canalicular pathology, (2) infection or tumor pathology, and (3) instrumented procedures.

### 2.2. Outcomes Measures

We evaluated preoperative data including age, sex, pain (Visual Analog Scale, VAS), disability (Oswestry Disability Index, ODI), preoperative neurology (neurogenic intermittent claudication and bladder-bowel symptoms) and confirmed radiologic diagnosis. Pain, disability and neurological findings were compared between postoperative day 1, 2 weeks, 6 months, and 12 months after the procedure. For comparisons of disability and satisfaction, we used the ODI and satisfaction ratings (excellent, good, fair, and poor).

### 2.3. Description of the Techniques

#### 2.3.1. Preoperative Considerations and Instruments

For preoperative planning, the spinal canal should be evaluated using magnetic resonance imaging (MRI). Radiologically, lateral recess stenosis and central stenosis are mainly targeted in bilateral traversing nerve root decompression. Herniated lumbar discs are targeted in the release of traversing nerve roots with the removal of protruded discs. For BESS, the interlaminar space and the location of the discs are checked using X-rays and computed tomography. Two measurements should be considered: (A) the distance between the upper and lower lamina and (B) the distance from the midline of the spine to the edge of the facet joint. The location for both the scope and instrument portals can be determined according to these lengths. We prepared each portal so that they were at least 3 cm wide to prevent interference with the instruments. The lower border of the upper lamina in the interlaminar space should be detached and used as an anatomical landmark. For adequate decompression of the traversing nerve root, the upper border of the lower lamina was also prepared with a radiofrequency coagulator. A saline irrigation system was used with a 0- or 12-degree endoscope, and a high-definition imaging system, a working channel, a standard laminectomy set, and a water correction system were employed.

#### 2.3.2. Patient Positioning

The patient is positioned on a radiolucent table with the C-arm located along the anterior-posterior axis to identify the interlaminar space and confirm the target level. An endoscopic screen is located directly opposite the surgeon, with the fluoroscopy screen located at the level of the patient’s hip.

#### 2.3.3. Initial Procedure Common to the Three Techniques Used

After identification of the interlaminar space, a serial dilator is used to progressively separate the relaxed muscle from the fascia. After placement of the scope and instrument portals, water irrigation is initiated. Bleeding is controlled by coagulation devices placed at the lower border of the upper lamina, medial border of the inferior articular process, and upper border of the lower lamina. The inferior part of the upper lamina, medial portion of the superior and inferior articular processes, and superior part of the lower lamina are removed using an electric drill and osteotome. The ligamentum flavum is safely dissected, released, and removed using pituitary forceps. The remnant bone and ligamentum flavum are trimmed and decompressed using a Kerrison punch, and bleeding is controlled using a tiny coagulator. Using an automated drill, unilateral laminectomy was performed until the upper margin of the ligamentum flavum was reached; during this procedure, the lower part of the spinal process was also widened to make space for contralateral laminectomy. Contralateral laminectomy was performed in a sublaminar fashion, with bony curettage and a small drill; sublaminar laminectomy was performed until the contralateral superior articular process was reached. After the partial removal of the contralateral facet, the lower and lateral margins were dissected. After dissection of the entire margin of the ipsilateral and contralateral ligamentum flavum, both were removed en bloc using pituitary forceps. In cases where discectomy was required, this was performed after dissection between the nerve and posterior longitudinal ligament. Using a scope retractor for protection, disc incision was performed with an Indian knife. Ruptured discs were removed using pituitary forceps or a hook dissector. In cases of calcified or hardened disc components, discectomy was performed using a Kerrison bone punch.

#### 2.3.4. Step-Ladder Technique

**Figure 1:**
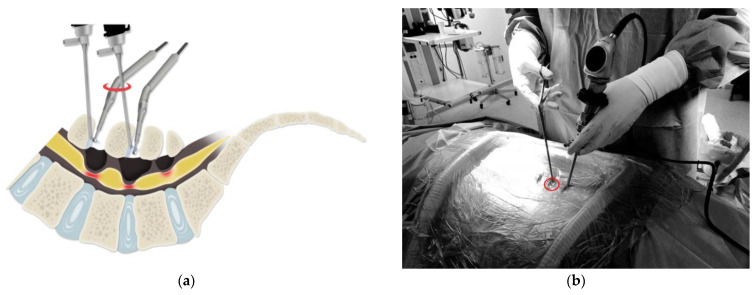

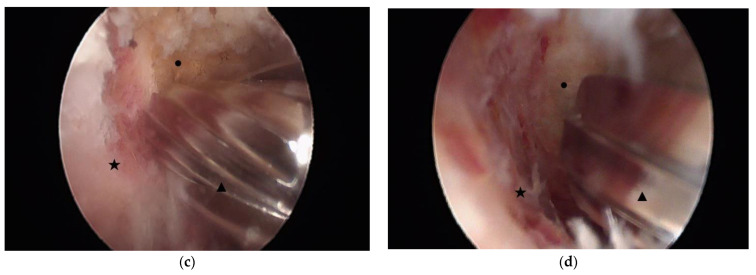
(**a**) Schematic illustration of the step-ladder technique. Sharing one portal decreases the number of incisions and risk of muscle injury (red Circle: Sharing portal). (**b**) Portal placement for the step-ladder technique. The scope portal (red circle, sharing portal), which is used during decompression of the first level, is changed to an instrument portal during decompression of the second level (red Circle: Sharing portal). (**c**) Endoscopic image of the lower laminectomy, showing that the lower level is more horizontal than the upper level (★: laminar, ●: yellow ligament, ▲: electric drill). (**d**) Endoscopic image of the upper-level laminectomy, showing that the upper level is more vertical than the lower level (★: laminar, ●: yellow ligament, ▲: electric drill).

The step-ladder technique was applied in cases with more than three levels or when long distances were present between laminae. After decompression of the first spinal level, the instrument can be removed, and the previous upper portal can be used as a lower portal for the second level (Figure 1a). Using the same incision, the scope is changed to a 40–60° scope relative to the upper level (Figure 1b). The interlaminar space and lower border of the upper lamina are detached under fluoroscopic guidance. Only one additional incision is made over the superior lamina. Two-level decompression laminectomy can be performed via three incisions using this technique. The angle of the procedure can be modified, as needed, through a common portal (Figure 1c,d).

#### 2.3.5. Portal Change Technique

**Figure 2:**
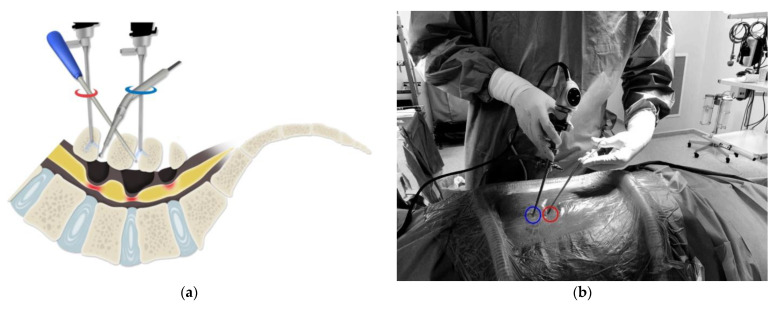

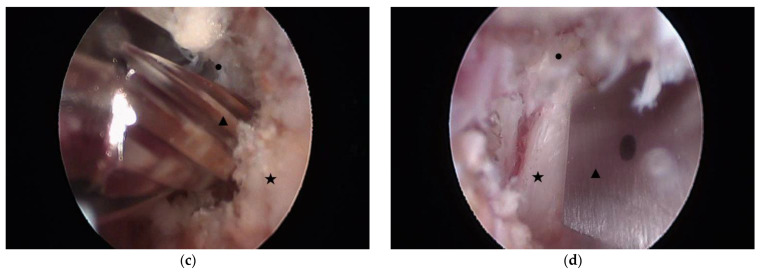
(**a**) Schematic illustration of the portal change technique. The sharing of two portals can decrease the number of portals required for the procedure. (**b**) Portal placement: decompression of the upper level is performed through the scope portal (red circle) and the instrument portal (blue circle), with decompression of the lower level performed through the instrument portal (red circle) and the scope portal (blue circle). (**c**) An endoscopic image of the upper-level laminectomy (Right L4-5) using an electric drill. (★: laminar, ●: yellow ligament, ▲: electric drill). (**d**) An endoscopic image of lower-level laminectomy (Right Lumbar 5-sacrum1) using an osteotome (★: laminar, ●: yellow ligament, ▲: osteotome).

We applied the portal change technique in cases of two-level laminectomy with a short distance between the levels. The portal technique can be described as upper-level laminectomy being performed through the 1st (highest) portal used as the endoscopic channel and lower-level laminectomy being performed through the 1st channel used as the instrumental channel and 2nd channel used as the endoscopic channel (Figure 2a). Each portal is placed over the upper lamina of the target level. The upper level is reached using a vertical endoscope, with decompression performed through the lower portal (Figure 2b). After initial level decompression, the orientation of the working channel and instrument portal is changed to perform decompression of the lower level. Specifically, the lower portal becomes the scope portal and the upper portal the instrument portal (Figure 2b). Lower-level decompression can be easily performed using an osteotome rather than an electric drill (Figure 2c). In a right-sided approach, upper-level decompression is performed via the lower portal and lower-level decompression via the upper portal (Figure 2d). Two-level decompression can be completed using only two portals with this technique.

#### 2.3.6. Tilting Technique

**Figure 3:**
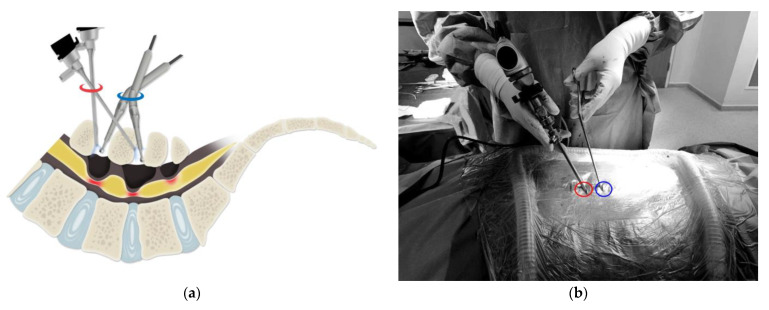

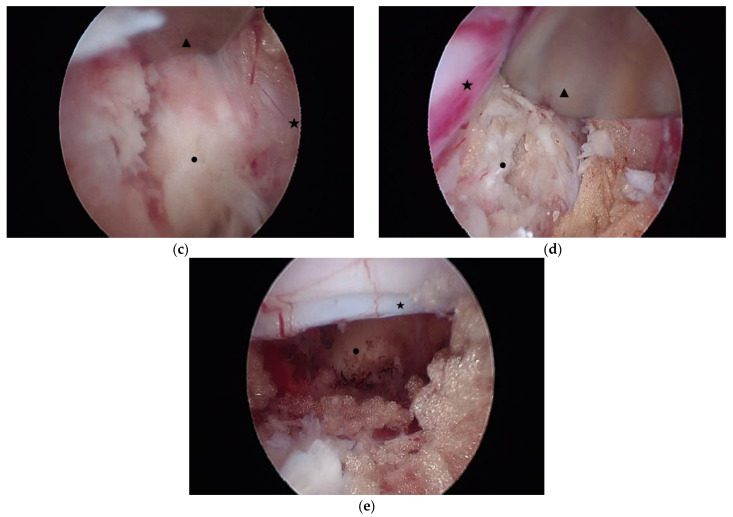
(**a**) Schematic illustration of the tilting technique. The sharing of the portals reduces the number of skin incisions required and preserves muscle tissue.(blue: scope portal, red: instrumental portal (**b**) Angle of tilt for both the scope and instrumental portal during upper-level laminectomy. This was an upper two-level case using the tilting technique with one more portal applied for the last level. (**c**) An endoscopic image of the middle level discectomy (lumbar 4–5), with the disc viewed directly (★: traversing nerve root, ▲: scope dural protector, ●: ruptured disc). (**d**) An endoscopic image of upper-level discectomy (lumbar 3–4), showing distortion of the disc space in the direct cranial direction (★: traversing nerve root, ▲: scope dural protector, ●: ruptured disc). (**e**) An endoscopic image of lower-level discectomy (lumbar 5-sacrum1), showing distortion of the disc space in the direct caudal direction (★: traversing nerve root, ●: ruptured disc); Blue circle: scope portal, Red circle: Instrumental potal.

The tilting technique is appropriate for patients with short lumbar height, tall patients, or those with dense muscles. This is a two-portal system. The upper portal should be located between the first and second lumbar laminar levels. The lower portal is located between the second and third lumbar laminar levels (Figure 3a,b). After decompression of the middle level, decompression of the upper level is performed by tilting both portals, with the lower portal tilted to a greater degree than the upper portal. The combination of the portal change technique and the tilting of the lower portal allows for decompression of the lower spinal level, with the removal of the spinal disc performed via the same portal. Therefore, the tilting technique allows for decompression of three spinal levels using only two portals, with the angle of tilt modified as necessary for appropriate orientation during the surgery (Figure 3c–e).

### 2.4. Statistical Analysis

Preoperative and postoperative (12 months) pain (VAS score) and disability (ODI) were compared with two-sided student *t*-test. R software for Windows (version 3.6.0; R Core Team; Vienna, Austria) was used, and statistical significance was set at *p*-values < 0.05.

## 3. Results

Of all the 132 cases of the laminectomy procedure performed using the BESS technique, 4 cases were not followed-up long-term. Six cases combined with the paraspinal approach were also excluded. The patient demographics, number of incisions, and clinical outcomes are summarized in Table 1. The mean age was 69.65 ± 8.24 years; the mean follow-up duration was 12.61 ± 1.78, and 55.74% and 44.26% of the participants were men and women, respectively.

**Figure 4:**
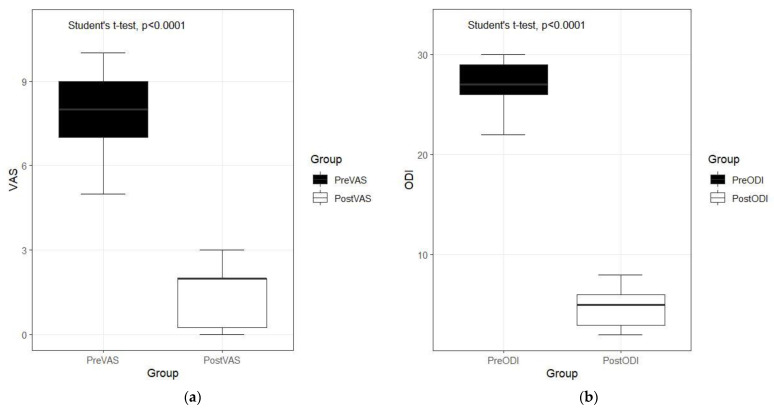
(**a**) Comparison of preoperative and postoperative pain improvement. (**b**) Comparison of preoperative and postoperative disability.

Most importantly, among the 264 levels of the procedure, a total of 580 incisions were noted; the mean incision per level was 1.44. Pain decreased significantly from 8.03 ± 1.42 to 4.77 ± 3.51, *p* < 0.001, Figure 4a). Disability improved significantly from 27.28 ± 1.78 to 4.93 ± 1.73 (*p* < 0.001, Figure 4b). Patient satisfaction in all the cases was 91.80 ± 13.42. There were four incidental durotomies identified. All cases were treated conservatively. No other complications such as infection were identified.

## 4. Discussion

To briefly summarize our findings, conventional BESS requires two incisions per level; however, this can be reduced to 1.44 incisions per level through various techniques.

Although open microscopic decompression is considered a standard technique for the treatment of multiple spinal stenosis [6,7], the outcomes from endoscopic techniques have improved with instrument developments combined with advancements in technique. Full endoscopic techniques (uniportal) and endoscopy-assisted techniques (biportal) possess both advantages and disadvantages. Basically, the uniportal technique can reduce the number of incisions and approach the interlaminar space directly, thereby minimizing tissue injury. However, a lack of muscle preparation and the small size of the cannulae, bone work, and removal of the ligamentum flavum are limitations of these techniques [8].

### 4.1. Technical Consideration of These Techniques

#### 4.1.1. Limitations of the Techniques

In the step-ladder technique, the lower portal is located at a lower level than usual. Consequently, the lower portal could be placed too low when using this technique on tall patients. Moreover, the portal change and tilting techniques cannot be performed in patients with a hyperlordotic spine as the angles cannot be appropriately corrected.

#### 4.1.2. Measures for Preventing Complications

Placement of a drainage bag can help prevent hematomas. Strict bleeding control can be accomplished by avoiding the use of an infusion pump [3], discontinuing antiplatelet therapy before surgery [2], or through the use of gel-foam or bone wax and the use of a coagulator. Although high-definition imaging can improve safety, the tilting and step-ladder techniques could result in incidental durotomy. Thus, the dissection of the dura and ligamentum flavum using curettage and a protector is warranted.

#### 4.1.3. Specific Perioperative Considerations (Pre- and Postoperative Workup; Postoperative Care Instructions)

Intra- and perioperative complications related to water irrigation and iatrogenic spondylosis are possible. Thus, chest and lumbar spine radiographs should be evaluated postoperatively.

#### 4.1.4. Specific Information to Be Provided to Patients Regarding Potential Risks of Surgery

Patients should be informed of the risk of hematoma and dural tearing.

#### 4.1.5. Summary of 10 Key Points

1. Techniques are indicated for the treatment of multilevel degenerative spinal pathology.
2. Pre-operative measurement of interlaminar and inter-spinous spaces is important.
3. Fluoroscopic guidance in the anterior-posterior directions is warranted.
4. Water irrigation is an important initial step for clear imaging and for creating a safe operative field.
5. The lower border of the upper lamina is a major landmark for BESS at every level.
6. With the step-ladder technique, the scope portal can be used as the instrument portal on subsequent levels.
7. For the portal change technique, both portals can be interchanged to perform decompression at additional spinal levels.
8. The tilting technique alters the angle of the scope and instrument portals, allowing for decompression at additional spinal levels without increasing the number of incisions.
9. Placement of a drainage bag can prevent a hematoma.
10. For the tilting and portal change techniques, dural protection can be ensured during dissection.

### 4.2. Clinical Outcome and Possible Applications

BESS has already shown efficacy in pain and disability improvement in several studies [9,10]. However, its superiority over other techniques is unclear. Upon comparison with the full endoscopic technique, less postoperative pain was observed following the full endoscopic technique; however, the long-term result was not statistically significant [11]. Upon comparison with open conventional techniques, although BESS resulted in a short hospital stay and better outcomes postoperatively, the long-term results were not very different [5,12]. The step-ladder technique is the most common technique, and is less challenging than other techniques. We applied multi-level techniques because BESS is effective in movable channel surgery [13]. Continuous irrigation ensures smooth water flow [14], which can serve as a better channel than creating a new channel. The portal change technique is considered the most challenging technique if the surgeon is unable to use both hands freely; however, it can be employed in clinical practice in procedures such as a laminectomy without flavectomy or discectomy; it can also be used to perform two-level surgeries. Moreover, in biportal spine fusion surgery, most L5–S1 procedures require left-hand operation for discectomy and cage placement [15]. The tilting technique is commonly employed in full endoscopic spine surgery; it has demonstrated efficacy as a multilevel procedure (more than three-level surgery) [16], with the ability to use more varied instruments [17] than the full endoscopic technique.

Similar to the findings of other studies on pain disability improvement [18], our result showed significant improvement. We achieved comparable laminectomy and en bloc ligamentum flavum removal, not piecemeal removal of ligamentum flavum or a foraminotomy procedure. Although not evaluated in the study, both the unilateral approach and bilateral decompression and lateral approach can be performed without the need for an additional incision. This can be studied in future studies. We achieved excellent postoperative results and good patient satisfaction using the techniques described in this study. As in other studies, the present results showed minor complications in incidental durotomy [19].

For better result for BESS, many surgeons strive to improve their instrument utilization [20] and expand the range of indications such as endoscopy-assisted screw fixation [21]. Exploring different angles for endoscopy can also be considered an option for better outcomes while preserving tissue [22]. Our findings are consistent with the notion that similar results can be obtained while preserving muscle tissue. Efforts for broadening the scope indications and prioritizing patient safety should be simultaneously pursued.

Although we aimed to introduce several techniques for reducing the number of incisions and preserving normal tissue, our study has several limitations. First, this is not a comparative study, so comparison between the experimental and control group was not performed. Secondly, this is a single-center study; thus, multicenter studies should be conducted to validate our findings. As this study focused on illustrating techniques, there are limitations in the clinical analysis and a detailed analysis of clinical outcomes is needed for the evaluation of the efficacy of these techniques.

## 5. Conclusions

The number of incisions can be reduced and the anatomical structure can be preserved using the step-ladder, portal change, and tilting techniques. These techniques also demonstrated improvements in pain, disability, and patient satisfaction.

## Figures and Tables

**Table 1:** Patient demographics and clinical outcomes in multi-level biportal spine surgery.

Criteria			
Age	69.65 ± 8.24		
Sex (*n*, %)	Female (68, 55.74%), Male (54, 44.26%)		
Follow up duration (month)	12.61 ± 1.78		
Surgical level and Incisions per levels	Surgical level: 264. Incision per levels (1.44, Total 580 incisions)		
Radiating pain (VAS)	Preoperative	Postoperative	*p*-value
	8.03 ± 1.42	4.77 ± 3.51	>0.001
Disability (Oswestry disability Index)	Preoperative	Postoperative	*p*-value
	27.28 ± 1.78	4.93 ± 1.73	>0.001
Patient satisfaction (Modified Macnab score)	91.80 ± 13.42		

VAS: Visual Analog Scale.

## Data Availability

Readers can request data from the corresponding author.
